# Associations of financial stressors and physical intimate partner violence perpetration

**DOI:** 10.1186/s40621-016-0069-4

**Published:** 2016-03-01

**Authors:** Laura M. Schwab-Reese, Corinne Peek-Asa, Edith Parker

**Affiliations:** 1 Department of Community & Behavioral Health, University of Iowa College of Public Health, Iowa City, IA 52240 USA; 2 Department of Occupational & Environmental Health, University of Iowa College of Public Health, Iowa City, IA 52240 USA; 3 Kempe Center for the Prevention and Treatment of Child Abuse and Neglect, University of Colorado, Anschutz Medical Campus, 13123 E 16th Ave, B390, Aurora, CO 80045 USA

**Keywords:** Financial stress, Stressors, Intimate partner violence, Perpetration

## Abstract

**Background:**

Contextual factors, such as exposure to stressors, may be antecedents to IPV perpetration. These contextual factors may be amenable to modification through intervention and prevention. However, few studies have examined specific contextual factors. To begin to address this gap, we examined the associations between financial stressors and three types of physical IPV perpetration.

**Methods:**

This analysis used data from Wave IV of The National Longitudinal Study of Adolescent to Adult Health. We used logistic regression to examine the associations of financial stressors and each type of IPV (minor, severe, causing injury), and multinomial logit regression to examine the associations of financial stressors and patterns of co-occurring types of IPV perpetration (*only* minor; *only* severe; minor and severe; minor, severe, and causing injury; compared with no perpetration).

**Results:**

Fewer men perpetrated threats/minor physical IPV (6.7 %) or severe physical IPV (3.4 %) compared with women (11.4 % and 8.8 %, respectively). However, among physical IPV perpetrators, a higher percentage of men (32.0 %) than women (21.0 %) reported their partner was injured as a result of the IPV. In logistic regression models of each type of IPV perpetration, both the number of stressors experienced and several types of financial stressors were associated with perpetrating each type of IPV. Utilities nonpayment, housing nonpayment, food insecurity, and no phone service were associated with increased odds of perpetrating each form of IPV in adjusted analysis. Eviction was associated with perpetrating severe physical IPV but not threats/minor IPV or IPV causing injury. In multinomial logit regression comparing patterns of IPV perpetration to perpetrating no physical IPV, the relationships of financial stressors were less consistent. Food insecurity was associated with perpetrating only minor physical IPV. Comparatively, overall number of financial stressors and four types of financial stressors (utilities nonpayment, housing nonpayment, food insecurity, and disconnected phone service) were associated with perpetrating all three forms of physical IPV.

**Conclusions:**

Combined with prior research, our results suggested interventions to improve financial well-being may be a novel way to reduce physical IPV perpetration.

## Background

Intimate partner violence (IPV), defined as psychological, physical, or sexual violence within the context of a current or former romantic relationship, is a significant threat to the health and well-being of the United States population (Coker et al. 2002). Up to a third of women and a quarter of men report experiencing physical or sexual assault by an intimate partner during their lifetime (Black et al. 2011). Victimization of IPV is associated with significant health burdens and has been linked to a number of psychological (Shorey et al. 2011; Beydoun et al. 2012; Próspero 2007; Próspero and Kim 2009; Okuda et al. 2011) and physical (Coker et al. 2000; Campbell et al. 2002; Carbone-López et al. 2006; Bonomi et al. 2007; Ellsberg et al. 2008; Straus et al. 2009) health consequences.

Despite a growing evidence base on the prevalence, risk factors, and intervention programs for IPV, much of the literature focuses on risk factors and intervention efforts for victims of IPV, with relatively little emphasis on perpetrators. As a result, there are few empirically supported interventions for IPV perpetrators (Eckhardt et al. 2006). Some researchers suggest that the limited effectiveness of prevention and intervention efforts is the result of the oversimplification of antecedents to perpetration, especially the contextual factors at the time of perpetration (Bell and Naugle 2008).

Stress, the individualized response to challenging situations, is associated with increased risk for IPV (Capaldi et al. 2012; Cano and Vivian 2001; Mason and Smithey 2012; Roberts et al. 2011). However, few studies have examined specific stressors, which are the events that trigger the stress response, as antecedents to IPV perpetration (Langhinrichsen-Rohling et al. 2012a; Elkins et al. 2013; Whitaker 2013; Byun 2012; Shortt et al. 2013). The Catalyst Model of Aggression hypothesized individuals develop a predisposition to violence as a result of personal characteristics (e.g., genes, personality) and historical environmental factors (e.g., exposure to violence) (Ferguson and Dyck 2012). Individuals with a high predisposition to violence are more likely to respond to environmental triggers (e.g., social interactions, stressors) with violence, compared with individuals with a lower predisposition to violence (Ferguson and Dyck 2012). Although untested for IPV perpetration, there is some evidence the Catalyst Model predicts other forms of violence perpetration (Ferguson et al. 2013; Ferguson et al. 2008b; Ferguson et al. 2008a).

Financial stress often impacts both individuals and the couple (Mason and Smithey 2012) and is a commonly cited antecedent to IPV perpetration (Byun 2012; Slep et al. 2010; Neff et al. 1995). However, IPV is not more prevalent in areas with low neighborhood income or high socioeconomic deprivation, which may be indicative of the financial stressors experienced by residents (Khalifeh et al. 2013; Bonomi et al. 2014). This difference in findings highlights the difference between stressors (the event) and stress (the response). Individual stress responses to environmental stressors vary as a result of a wide range of factors, such as genes (Bouma et al. 2012) and prior experiences (Lovallo 2013), so the relationship between financial context and IPV perpetration may depend on the distinction between financial stress and stressors (Benson et al. 2003). Perceptions of financial stress (individuals’ processing of stressor events), are significantly associated with IPV victimization among women, but more objective measures of financial stressors (the event which causes the stress response), such as income to needs ratio, are not significantly associated with IPV victimization (Benson et al. 2003). Additionally, perceived financial stress is a significant predictor of physical IPV perpetration among both men and women in two studies (Neff et al. 1995; Slep et al. 2010). To our knowledge, there have been no studies of the relationship between specific financial stressors and IPV perpetration so it is unclear if exposure to financial stressors is an antecedent to IPV perpetration.

The purpose of this analysis is to determine the extent to which experiences of specific financial stressors are associated with making threats of violence/minor physical IPV perpetration, severe physical IPV perpetration, and physical IPV resulting in injury, as reported by perpetrators. Given the limited effectiveness of IPV prevention and intervention efforts, it is important to understand modifiable risk factors, such as stressors, and their relationship with IPV perpetration. The results of this analysis may have important implications for the development and implementation of IPV prevention and intervention activities, especially those targeting social context at the time of IPV perpetration.

## Methods

We performed a secondary analysis of the Wave IV data of The National Longitudinal Study of Adolescent to Adult Health (Add Health). The Add Health study began in 1994 and continued through 2008 with four waves of data collection (Harris et al. 2009; 2013). The study design included systematic sampling methods and stratification to ensure the sample was representative of the United States population of adolescents (Harris et al. 2009; 2013). Of the 20,745 participants who enrolled in the study, 15,701 were interviewed during the 2008 Wave IV when they were between the ages of 24 and 32 years (Harris et al. 2009; 2013). Additional information on the study design, including information on the institutional review board approval and informed consent process, is available elsewhere (Harris et al. 2009; 2013).

To be eligible for this analysis, participants must have been in a relationship during the past year. IPV perpetration was measured as occurring within the last 12-months of the most recent relationship. Since measurement of exposure to financial stressors was limited to the 12 months prior to the interview, participants who reported on relationship experiences more distant than the prior 12 months were excluded. Additionally, participants who were incarcerated during the interview were excluded because the outcomes for this analysis required the ability to have physical contact with an intimate partner. Participants were also excluded if they did not respond to questions about IPV perpetration, financial stressors, or study covariates.

A total of 15,701 participants completed data collection during Wave IV of the Add Health study. Of these participants, 3320 (21.1 %) were excluded because they were not in a relationship during the past twelve months and/or were currently incarcerated. Of the remaining 12,381 participants, 882 (7.1 %) were excluded due to missing data for IPV questions, financial stressors, or other covariates, resulting in a sample size of 11,499. As recommended by the Add Health research team, we set the weight of any excluded participants to 0.000001, which ensures the weighting process is correctly completed, but excluded participants do not contribute to the estimates produced by the models (Chen and Chantala 2014). Dropping participants from the weighting protocol would result in unrepresentative weights and incorrect standard errors (Chen and Chantala 2014).

### Measurement

#### Intimate partner violence perpetration

The outcomes of this analysis were three forms of physical IPV: making threats of physical IPV/minor physical IPV (“How often have you threatened [partner] with violence, pushed or shoved (him/her), or thrown something at (him/her) that could hurt?); severe physical IPV (How often have you slapped, hit, or kicked [partner]?); and physical IPV resulting in injury (How often has [partner] had an injury, such as a sprain, bruise, or cut because of a fight with you?) (Harris et al. 2009; 2013).

Participants reported how frequently they perpetrated each form of IPV on a scale ranging from “never” to “more than twenty times in the last year”. Each question was dichotomized to categorize participants as perpetrating this form of IPV at least once in the prior year or not perpetrating this form. Using these questions, we created mutually exclusive categorizations of the patterns of IPV perpetration, which were used in the multinomial models: only threats/minor physical; only severe physical; threats/minor physical and severe physical; and all three forms including physical resulting in injury. Thirty-two participants (0.2 %) reported perpetrating severe physical IPV and IPV resulting in an injury but not threats/minor IPV. Given the small number, these participants were excluded from the multinomial models of patterns of IPV but were included in all other analyses.

#### Financial stressors

Participants reported if they had experienced six types of financial stressors at least once in the past twelve months: nonpayment of utilities for financial reasons, nonpayment of housing for financial reasons, fears of food unavailability, phone disconnected due to financial reasons, utilities turned-off due to nonpayment, and eviction from housing (Harris et al. 2009; 2013).

We summed the total number of financial stressors experienced by each participant, which ranged from zero (no financial stressors in past year) to six (all forms of financial stressors in the past year), which is consistent with prior treatment of this measure (Halliday Hardie and Lucas 2010). We created models using both the total number of financial stressors and each individual type of financial stressor. Additional information on the treatment of the exposure and outcome variables is included in Appendix.

#### Covariates

We considered a range of possible covariates with relationships to IPV perpetration and/or financial stressors. We selected our covariates based on a literature review and a directed acyclic graph (DAG) (Shrier and Platt 2008), a visual representation of the assumed relationships between the variables of interest and their covariates, which allows for careful selection of covariates to avoid introducing bias through covariate selection. Selected covariates included gender, race, health, neuroticism, alcohol abuse, and drug abuse. We also considered controlling for perceived stress and relationship conflict, but conceptualized those variables as part of the causal pathway between financial stressors and IPV perpetration. We also considered controlling for education and employment, but felt that the potential bias of those variables was, in our conceptualization, controlled through the selected covariates.

Gender and race were measured using participant self-report. Perceived health was measured as participant self-reported general health. Neuroticism was measured using the NEO-FFI scale (Harris et al. 2009), which summed responses to twelve questions to create a single measure of neuroticism. Alcohol abuse was measured as self-reported experience of the diagnostic criteria for alcohol dependence or abuse as defined by the *Diagnostic and Statistical Manual of Mental Disorders* (DSM-IV) (American Psychological Association 1994) such as increased tolerance, withdrawal symptoms, and/or social or interpersonal issues related to alcohol use. Drug abuse was measured using the number of self-reported symptoms consistent with DSM-IV diagnostic criteria for drug abuse, including failure to perform role obligations, legal problems, and/or social or interpersonal issues associated with drug use (American Psychological Association 1994).

### Statistical analysis

All analyses were weighted and clustered by school and primary sampling unit to account for the survey design of the Add Health study as recommended by the Carolina Population Center (Chen and Chantala 2014). All descriptive analyses reported the weighted point estimates and 95 % confidence intervals for the estimates. Chi-square tests were used to determine if exposure to financial stressors and rates of IPV perpetration were equal across gender (Table 1).

Weighted, clustered, single-variable and multivariable regression models were used to estimate the association between each financial stressor, the total number of financial stressors, and IPV perpetration. IPV perpetration was modeled two ways. First, we modeled each form of IPV separately in logistic regression, which models the odds of perpetrating the specific form of IPV compared with participants who did not perpetrate that form of IPV (Table 2). In this analysis, categorizations were not mutually exclusive and may have resulted in substantial heterogeneity of the comparison group, because it included participants who perpetrated no forms of IPV but also those who perpetrated other forms of IPV. To reduce this heterogeneity, we used multinomial logit regression models, which modeled IPV as mutually exclusive categorizations of perpetration compared to no IPV perpetration (Table 3). Although the first method of analysis is more common in the literature, multinomial logit regression models may be a more precise method of analysis when multiple outcomes co-occur (Fulu et al. 2013).

We also examined gender as a possible modifier of the relationship between financial stressors and IPV perpetration. We assessed moderation by including an interaction term of gender and the exposure variable in the final models and examining the significance of the interaction term.

## Results

### Descriptive statistics

Significantly more women (27.7 %) than men (22.9 %) experienced at least one financial stressor (Table 1). A higher percentage of women than men reported experiencing three of the six types of financial stressors. A significantly higher number of women than men were unable to pay their utilities (17.6 % vs 12.7 %), reported food insecurity (14.0 % vs 9.9 %), and experienced disconnected phone service (10.4 % vs 7.8 %). Men and women experienced housing nonpayment, having utilities turned-off, and eviction in approximately the same proportions.

**Table 1:** Estimated proportion and 95 % confidence estimates of exposure to financial stressors and perpetration of three forms of physical IPV

	Men	Women	Difference
	Percent (95 % CI)		P-value^a^
Financial stressors			
Number of financial stressors			<.0001
None	77.2 (75.4–79.1)	72.3 (70.2–74.5)	
1–2	16.3 (14.2–18.4)	18.8 (16.8–20.8)	
3–4	5.2 (4.1–6.2)	7.3 (6.0–8.6)	
5–6	1.4 (0.9–1.8)	1.5 (1.0–2.0)	
Utilities nonpayment			
Yes	12.7 (11.5–14.0)	17.6 (15.7–19.4)	<.0001
No	87.3 (86.0–88.5)	82.4 (80.6–84.3)	
Housing nonpayment			
Yes	9.4 (8.4–10.4)	10.2 (9.0–11.3)	.26
No	90.6 (89.6–91.6)	89.8 (88.7–91.0)	
Food insecurity			
Yes	9.9 (8.7–11.1)	14.0 (12.6–15.3)	<.0001
No	90.1 (88.9–91.3)	86.0 (84.7–87.4)	
No phone service			
Yes	7.8 (6.8–8.8)	10.4 (9.3–11.5)	<.0001
No	92.2 (91.2–93.2)	89.6 (88.5–90.7)	
Utilities turned off			
Yes	5.4 (4.6–6.3)	5.4 (4.6–6.1)	.82
No	94.6 (93.7–95.4)	94.7 (93.9–95.4)	
Evicted			
Yes	1.0 (0.7–1.4)	1.0 (0.7–1.4)	.93
No	99.0 (95.6–99.3)	98.9 (98.6–99.3)	
Intimate partner violence perpetration			
Threats/Minor physical			
Yes	6.7 (5.7–7.5)	11.4 (10.1–12.7)	<.0001
No	93.4 (92.5–94.3)	88.6 (87.3–89.9)	
Severe physical			
Yes	3.4 (2.9–4.0)	8.8 (7.8–9.9)	<.0001
No	96.6 (96.0–97.2)	91.2 (90.1–92.2)	
If Yes to physical, caused injury			
Yes	32.0 (25.6–38.5)	21.0 (17.7–24.4)	.003
No	68.0 (61.5–74.4)	79.0 (75.6–82.3)	

^a^Chi-square test

A significantly higher number of women reported perpetrating threats/minor physical IPV (11.4 % vs 6.7 %) and severe physical IPV (8.8 % vs 3.4 %). However, among perpetrators of physical IPV, a significantly higher number of men reported causing injury to their partner (32.0 % vs 21.0 %). Overall, 92.9 % (95 % CI: 92.0–93.8) of men and 86.7 % (95 % CI: 85.5–88.2) of women did not perpetrate any form of IPV in the prior year.

### Intimate partner violence and financial stressors

We did not find evidence to support significant moderation of the relationship between financial stressors and IPV perpetration by gender. Since stratification did not significantly influence the results, we report only the combined results. In short, although women experience both IPV perpetration and financial stressors at a higher rate, the associations of financial stressors and IPV perpetration were not significantly different between men and women.

Overall, the number of financial stressors was strongly associated with each form of physical IPV, after adjusting for gender, race, perceived health, neuroticism, alcohol use, and drug use (Table 2). For each additional financial stressor, the odds of perpetration increased 1.16 times (95 % CI: 1.09–1.23) for threats/minor physical IPV, 1.22 times (95 % CI: 1.14–1.30) for severe physical IPV, and 1.27 times (95 % CI: 1.16–1.38) for IPV resulting in injury.

**Table 2:** Association of financial stressors and perpetration of threats of IPV, physical IPV, and IPV resulting in injury

	Threats/Minor physical IPV^a^		Severe physical IPV^b^		IPV resulting in injury^c^	
	Crude OR (95 % CI)	Adjusted OR (95 % CI)^d^	Crude OR (95 % CI)	Adjusted OR (95 % CI)^d^	Crude OR (95 % CI)	Adjusted OR (95 % CI)^d^
Continuous measure of number of stressors						
Number of financial stressors^e^	1.29 (1.22–1.36)	1.16 (1.09–1.23)	1.34 (1.26–1.44)	1.22 (1.14–1.30)	1.42 (1.30–1.54)	1.27 (1.16–1.38)
Individual stressors						
Utilities nonpayment^f^	1.84 (1.53–2.23)	1.37 (1.13–1.65)	2.08 (1.67–2.60)	1.54 (1.23–1.92)	2.42 (1.75–3.35)	1.76 (1.28–2.43)
Housing nonpayment^f^	2.10 (1.69–2.61)	1.68 (1.33–2.12)	2.17 (1.74–2.71)	1.74 (1.38–2.20)	2.35 (1.64–3.36)	1.78 (1.21–2.59)
Food insecurity^f^	2.24 (1.84–2.74)	1.56 (1.28–1.90)	2.36 (1.81–3.07)	1.64 (1.25–2.15)	3.61 (2.51–5.20)	2.47 (1.71–3.58)
No phone service^f^	2.10 (1.70–2.58)	1.48 (1.21–1.83)	2.78 (2.21–3.51)	2.04 (1.61–2.59)	3.22 (2.21–4.69)	2.19 (1.49–3.20)
Utilities turned off^f^	1.68 (1.30–2.17)	1.26 (0.94–1.69)	1.75 (1.30–2.35)	1.35 (0.99–1.84)	2.05 (1.35–3.12)	1.49 (0.93–2.25)
Evicted^f^	2.37 (1.46–3.85)	1.68 (0.98–2.88)	3.31 (1.88–5.82)	2.39 (1.35–4.23)	0.95 (0.30–3.04)	0.61 (0.19–2.02)

^a^Compared to those who did not perpetrate threats/minor physical IPV

^b^Compared to those who did not perpetrate severe physical IPV

^c^Compared to those who did not perpetrate IPV resulting in injury

^d^Adjusted for gender, race, perceived health, neuroticism, alcohol abuse or dependence, and drug abuse

^e^Modeled as continuous ranging from 0 to 6 forms of financial stressors

^f^Ref = No experience of this form of financial stressor

Four of the six types of financial stressor were associated with significantly higher odds of perpetrating each form of physical IPV compared to all others in the sample. In adjusted analysis, utilities nonpayment, housing nonpayment, food insecurity, and disconnected phone service were associated with increased odds of each form of physical IPV. In addition, the odds of perpetrating severe physical IPV were 2.39 times higher (95 % CI: 1.35–4.23 %) among those who were evicted compared to those who were not evicted.

In the remaining analysis, individuals who perpetrated each specific pattern of IPV were compared to those who perpetrated no forms of IPV (Table 3). In adjusted analyses, an increasing number of financial stressors was not associated with significantly increased odds of perpetrating only threats/minor physical IPV compared with perpetrating no IPV. However, the odds of perpetrating only severe physical IPV (OR: 1.20; 95 % CI:1.05–1.38), both threats/minor and severe physical IPV (OR: 1.19; 95 % CI: 1.08–1.32), and for all three forms of IPV (OR: 1.29; 95 % CI:1.18–1.41) significantly increased with each additional financial stressor, compared to individuals who did not perpetrate IPV.

**Table 3:** Association of financial stressors and patterns of IPV perpetration

	Only threats/minor physical^a^		Only severe physical IPV^a^		Threats/Minor & severe physical IPV^a^		All three forms^a^	
	Crude OR (95 % CI)	Adjusted OR (95 % CI)^b^	Crude OR (95 % CI)	Adjusted OR (95 % CI)^b^	Crude OR (95 % CI)	Adjusted OR (95 % CI)^b^	Crude OR (95 % CI)	Adjusted OR (95 % CI)^b^
Continuous measure of number of stressors								
Number of financial stressors^c^	1.19 (1.09–1.29)	1.07 (0.98–1.17)	1.27 (1.10–1.46)	1.20 (1.05–1.38)	1.33 (1.21–1.46)	1.19 (1.08–1.32)	1.44 (1.32–1.58)	1.29 (1.18–1.41)
Individual stressors								
Utilities nonpayment^d^	1.52 (1.18–1.97)	1.19 (0.91–1.55)	1.98 (1.28–3.05)	1.63 (1.06–2.49)	2.09 (1.53–2.84)	1.49 (1.10–2.03)	2.28 (1.63–3.19)	1.64 (1.17–2.31)
Housing nonpayment^d^	1.66 (1.21–2.28)	1.35 (0.97–1.89)	1.63 (0.94–2.80)	1.43 (0.84–2.43)	2.42 (1.75–3.34)	1.95 (1.40–2.71)	2.55 (1.75–3.70)	1.95 (1.61–2.91)
Food insecurity^d^	1.89 (1.43–2.50)	1.38 (1.05–1.82)	1.56 (0.92–2.65)	1.27 (0.75–2.15)	1.99 (1.39–2.84)	1.31 (0.91–1.88)	3.67 (2.47–5.44)	2.48 (1.64–3.73)
No phone service^d^	1.44 (1.01–2.05)	1.05 (0.74–1.49)	3.16 (1.90–5.24)	2.79 (1.69–4.60)	2.29 (1.63–3.22)	1.61 (1.14–2.27)	3.65 (2.45–5.43)	2.48 (1.67–3.70)
Utilities turned off^d^	1.37 (0.88–2.15)	1.04 (0.63–1.70)	1.04 (0.50–2.20)	0.94 (0.44–2.03)	1.83 (1.20–2.80)	1.41 (0.91–2.20)	2.23 (1.41–3.52)	1.56 (0.97–2.49)
Evicted^d^	1.22 (0.55–2.70)	0.86 (0.35–2.10)	3.11 (0.74–13.03)	2.23 (0.42–11.75)	5.00 (2.53–9.88)	3.72 (1.83–7.57)	1.28 (0.40–4.00)	0.84 (0.26–2.69)

^a^Compared to participants who perpetrated no forms of IPV in the past year

^b^Adjusted for gender, race, perceived health, neuroticism, alcohol abuse or dependence, and drug abuse

^c^Modeled as continuous ranging from 0 to 6 forms of financial stressors

^d^Ref = No experience of this form of financial stressor

The relationships between individual financial stressors and IPV perpetration were not consistent across patterns of perpetration, although more severe patterns of violence were associated with more of the individual financial stressors. In adjusted analysis, only food insecurity remained significantly associated with perpetration of only threats/minor physical IPV compared to perpetrating no IPV. Comparatively, utilities nonpayment, housing nonpayment, food insecurity, and disconnected phone service were significantly associated with perpetrating all three forms of physical IPV compared with individuals who perpetrated no physical IPV.

## Discussion

As hypothesized by the Catalyst Model of Aggression, exposure to stressors, specifically financial stressors, was associated with physical IPV perpetration among men and women. Consistent with the prior literature on the inequalities in wage and wealth acquisition by gender (Autor et al. 2008; Ruel and Hauser 2013) and IPV perpetration by gender (Langhinrichsen-Rohling et al. 2012b), a higher proportion of women reported experiencing financial stressors and perpetrating physical IPV. With few exceptions, both the number of financial stressors and the individual financial stressors were associated with increased odds of making threats/minor physical IPV, severe physical IPV, and IPV resulting in injury. There were some differences in these relationships when we examined specific patterns of perpetration. For example, the overall number of financial stressors was a significant predictor of threats/minor physical IPV perpetration when modeled as anyone who had perpetrated this form of IPV compared with all other participants, including perpetrators of other types of IPV. However, the overall number of financial stressors was not a significant predictor of threats/minor physical IPV perpetration when modeled as people who *only* perpetrated threats/minor IPV compared with individuals who perpetrated no IPV. This result suggests that exposure to financial stressors did not increase the odds of only threats/minor physical IPV perpetration. It did, however, increase the odds of this form of IPV in combination with other forms. These divergent findings have significant implications for the measurement and analysis of IPV perpetration. Although much of the literature has reported the results of separate models for each type of IPV outcome, the difference in findings between the two methods may support the use of multinomial models when predicting overlapping outcomes, such as multiple forms of IPV.

An unexpected finding was the overall lack of association between more severe forms of financial stressors and IPV perpetration. For example, in the multinomial model, eviction was not associated with perpetration of only threats/minor physical IPV, only severe physical IPV, or all three forms. Eviction was only associated with perpetration of both threats/minor and severe physical IPV. Similarly, having the utilities turned off was not associated with any of the patterns IPV perpetration, when compared to individuals who perpetrated no forms of physical IPV. This finding was unexpected because prior research suggested financial issues related to housing are more strongly associated with physical and mental manifestations of stress than food insecurity (Liu et al. 2014). Additionally, individuals who experienced moderate levels of housing strain (i.e., at least one late or incomplete payment) have reported substantially lower levels of psychological and physical distress compared with individuals who have been removed from their home due to nonpayment (Cannuscio et al. 2012). Additional research is necessary to further examine if the limited association between severe financial stressors and IPV perpetration is limited to this dataset, if it is a function of examining individual financial experiences rather than patterns of experiences, or if other factors may be contributing to this relationship.

Our results were, overall, consistent with the small body of literature connecting experience with financial stress to IPV. Our literature review identified nine studies on the associations between financial context and IPV perpetration. Three of those studies focused exclusively on male-perpetrated IPV as reported by the female victims (Bonomi et al. 2014; Byun 2012; Golden et al. 2013). Economic abuse, defined as behaviors that control a partner’s ability to be economically secure, is common among women who experience other forms of abuse (Adams et al. 2008). In addition, economic abuse may alter women’s perceptions of financial stress, so those results were not directly comparable to the results of this study (Postmus et al. 2011; Matjasko et al. 2013). However, each of these studies contributed to our general understanding of the role of financial stress in IPV perpetration.

One additional study examined the association of socioeconomic deprivation and IPV victimization among men and women (Khalifeh et al. 2013). Low social class, measured as the occupational classification of respondent, and low income were strongly associated with lifetime experiences of physical IPV among women but not among men (Khalifeh et al. 2013). However, these results must be interpreted with caution because it was unknown if current economic status was consistent with economic status at the time of the IPV victimization.

Two studies examined the association between financial stress and IPV as reported by the perpetrator. In a sample of men and women from Texas, perceived financial stress was a significant predictor of physical IPV perpetration among both men and women after adjusting for income, traditional beliefs about gender roles, and perpetrator and victim alcohol use (Neff et al. 1995.) In a study of men and women enlisted in the US Air Force, financial stress was also associated with physical IPV perpetration among both men and women (Slep et al. 2010). Generally, our findings were similar to these two studies, but we further examined the role of specific types of financial stressors, rather than overall perception of financial stress. Additionally, we expanded upon these findings to include multiple forms of physical IPV perpetration and found financial stressors were, overall, not associated with *only* making threats of physical IPV or minor physical IPV. However, overall number of stressors and individual types of stressors were significantly associated with more severe forms of IPV perpetration.

Finally, one study focused on male perpetrated IPV and included reports of both IPV and financial stress by both partners (Benson et al. 2003). In this study, subjective financial stress was strongly associated with increased odds of male-to-female physical IPV, but income-to-needs ratio, a more objective measure of financial stressors, was not associated with IPV perpetration. Our analysis was unable to measure perceptions of financial stress, defined as how individuals mentally respond to financial stressors, but it did find that experiencing financial stressors was associated with increased odds of IPV perpetration among men and women. The discrepancy between our findings and the findings on income-to-needs ratio may have important implications for intervention strategies because it may indicate that subjective financial stress and management of finances are better predictors of IPV perpetration than overall financial resources. Additional research examining the relative contributions of financial stress, financial stressors, and financial management may provide further guidance on effective financial interventions.

### Implications for intervention

In combination with prior findings, our findings have implications for the development of interventions to prevent IPV through improving the financial context of couples. Among female victims of IPV, career counseling has been shown to improve perceptions of financial well-being (Chronister et al. 2011; Davidson et al. 2012), however, it is not clear if career counseling would be effective to reduce exposure to financial stressors. Direct economic intervention (i.e., providing funds) may be another method of IPV prevention (Matjasko et al. 2013; Kim et al. 2007). However, a systematic review of economic interventions in low-income countries found mixed results in reducing IPV (Yvas and Watts 2009) so additional research is necessary before this type of intervention is implemented.

Our findings suggest less severe forms of financial stressors (e.g., utilities nonpayment) have a stronger association with IPV perpetration than the more severe forms of financial stressors (e.g., utilities turned off). However, individuals often experience the less severe forms of financial stressors prior to experiencing the more severe forms so interventions may effectively prevent IPV perpetration if they target individuals in the early stages of financial stressor experiences.

Future interventions may be guided by additional research into the differing relationships of IPV perpetration and perceptions of financial stress, specific financial stressors, and overall financial resources. Disentangling the relative contributions of financial stress resulting from poor financial management compared with financial stress resulting from limited financial resources may provide additional guidance into the most effective intervention methods. The development of future interventions may also be enhanced by additional information on the financial experiences of perpetrators. Our literature review suggests victims and their experiences are often the focus of this line of research so additional research on the financial experiences of victims, perpetrators, and victim-perpetrators may further guide the development and tailoring of effective interventions.

### Limitations

We relied upon self-report of IPV experiences by the perpetrator. A recent study suggests the reported rates of IPV vary significantly if IPV is measured based on reports by only the perpetrator, the victim, or both, so including reports of IPV by both partners would improve the study design (Renner et al. 2015). Additionally, our analysis is at the level of the individual, which limits our ability to make temporal inferences about the experiences of financial stressors and IPV perpetration. Since exposure to financial stressors may change over time, conducting an analysis at the event-level may be a better method of determining the relationship between financial stressors and IPV perpetration. For example, a recent study of the association between anger and IPV perpetration asked participants to complete daily diaries of their affect and their experiences with their partner (Elkins et al. 2013). By collecting data every day, the researchers were better able to determine the temporal association between the two factors. Another possible limitation is related to our treatment of the financial stressor variables. Our analysis examined number and type of financial stressors and did not examine patterns of stressors. As these stressors may occur simultaneously, additional research on the co-occurrence of multiple forms of financial stressors and relationships of patterns of co-occurring stressors with IPV perpetration may further enhance our understanding of these relationships. Finally, our analysis used secondary data, which limited operationalization of constructs to the variables collected in the study.

## Conclusions

Financial stressors are associated with physical IPV perpetration, but the relationship between specific financial stressors and IPV perpetration varies by the type of IPV perpetrated. These findings suggest intervention and prevention programs to reduce financial stressors may be a novel way to reduce future physical IPV perpetration.

**Table 4:** Definition of exposure and outcome variables

Variable	Definition	Variable type
Logistic regression outcome variables		
Making threats of physical IPV/minor physical IPV	How often have you threatened [partner] with violence, pushed or shoved (him/her), or thrown something at (him/her) that could hurt?	Dichotomous
Severe physical IPV	How often have you slapped, hit, or kicked [partner]?	Dichotomous
Physical IPV resulting in injury	How often has [partner] had an injury, such as a sprain, bruise, or cut because of a fight with you?	Dichotomous
Multinomial regression outcome variables		
Patterns of IPV perpetration	All possible mutually exclusive categories from the three logistic regression outcomes variables	Categorical
Exposure variables		
Utilities nonpayment	In the past 12 months, was there a time when {YOU/YOUR HOUSEHOLD} didn’t pay the full amount of a gas, electricity, or oil bill because you didn’t have enough money?	Dichotomous
Housing nonpayment	In the past 12 months, was there a time when {YOU/YOUR HOUSEHOLD} didn’t pay the full amount of the rent or mortgage because you didn’t have enough money?	Dichotomous
Food insecurity	In the past 12 months, was there a time when {YOU/YOUR HOUSEHOLD WERE/WAS} worried whether food would run out before you would get money to buy more?	Dichotomous
No phone service	In the past 12 months, was there a time when {YOU/YOUR HOUSEHOLD} was without phone service because you didn’t have enough money?	Dichotomous
Utilities turned off	In the past 12 months, was there a time when {YOU/YOUR HOUSEHOLD} had the service turned off by the gas or electric company, or the oil company wouldn’t deliver, because payments were not made?	Dichotomous
Evicted	In the past 12 months, was there a time when {YOU/YOUR HOUSEHOLD} were evicted from your house or apartment for not paying the rent or mortgage?	Dichotomous
Number of financial stressors	Summed total of six possible financial stressors	Continuous

## References

[CR1] Adams AD, Sullivan CM, Bybee D, Greeson MR (2008). Development of the scale of economic abuse. Violence Against Women.

[CR2] American Psychological Association (1994). Diagnostic and statistical manual of mental disorders (DSM).

[CR3] Autor DH, Katz LF, Kearney MS (2008). Trends in U.S. wage inequality: Revising the revisionist. Rev Econ Stat.

[CR4] Bell KM, Naugle AE (2008). Intimate partner violence theoretical considerations: moving towards a contextual framework. Clin Psychol Rev.

[CR5] Benson ML, Fox GL, DeMaris A, Van Wyk J (2003). Neighborhood disadvantage, individual economic distress and violence against women in intimate relationships. J Quant Criminol.

[CR6] Beydoun HA, Beydoun MA, Kaufman JS, Lo B, Zonderman AB (2012). Intimate partner violence against adult women and its association with major depressive disorder, depressive symptoms and postpartum depression: a systematic review and meta-analysis. Soc Sci Med.

[CR7] Black M, Basile K, Breiding M, Smith S, Walthers S, Merrick M (2011). The National Intimate Partner and Sexual Violence Survey (NISVS): 2010 summary report.

[CR8] Bonomi AE, Anderson ML, Rivara FP, Thompson RS (2007). Health outcomes in women with physical and sexual intimate partner violence exposure. J Womens Health.

[CR9] Bonomi AE, Trabert B, Anderson ML, Kernic MA, Holt VL (2014). Intimate partner violence and neighborhood income a longitudinal analysis. Violence Against Women.

[CR10] Bouma EMC, Riese H, Doornbos B, Ormel J, Oldehinkel AH (2012). Genetically based reduced MAOA and COMT functioning is associated with the cortisol stress response: a replication study. Mol Psychiatry.

[CR11] Byun S-H (2012). What happens before intimate partner violence? Distal and proximal antecedents. J Fam Violence.

[CR12] Campbell J, Jones AS, Dienemann J, Kub J, Schollenberger J, O’Campo P (2002). Intimate partner violence and physical health consequences. Arch Intern Med.

[CR13] Cannuscio CC, Alley DE, Pagán JA, Soldo B, Krasny S, Shardell M (2012). Housing strain, mortgage foreclosure, and health. Nurs Outlook.

[CR14] Cano A, Vivian D (2001). Life stressors and husband-to-wife violence. Aggress Violent Behav.

[CR15] Capaldi DM, Knoble NB, Shortt JW, Kim HK (2012). A systematic review of risk factors for intimate partner violence. Partner Abuse.

[CR16] Carbone-López K, Kruttschnitt C, Macmillan R (2006). Patterns of intimate partner violence and their associations with physical health, psychological distress, and substance use. Public Health Rep.

[CR17] Chen P, Chantala K (2014). Guidelines for analyzing add health data.

[CR18] Chronister KM, Harley E, Aranda CL, Barr L, Luginbuhl P. Community-based career counseling for women survivors of intimate partner violence: a collaborative partnership. J Career Dev. 2011; doi: 10.1177/0894845311401618.

[CR19] Coker AL, Smith PH, Bethea L, King MR, McKeown RE (2000). Physical health consequences of physical and psychological intimate partner violence. Arch Fam Med.

[CR20] Coker AL, Davis KE, Arias I, Desai S, Sanderson M, Brandt HM (2002). Physical and mental health effects of intimate partner violence for men and women. Am J Prev Med.

[CR21] Davidson MM, Nitzel C, Duke A, Baker CM, Bovaird JA (2012). Advancing career counseling and employment support for survivors: an intervention evaluation. J Couns Psychol.

[CR22] Eckhardt CI, Murphy C, Black D, Suhr L (2006). Intervention programs for perpetrators of intimate partner violence: conclusions from a clinical research perspective. Public Health Rep.

[CR23] Elkins SR, Moore TM, McNulty JK, Kivisto AJ, Handsel VA (2013). Electronic diary assessment of the temporal association between proximal anger and intimate partner violence perpetration. Psychol Violence.

[CR24] Ellsberg M, Jansen HA, Heise L, Watts CH, Garcia-Moreno C (2008). Intimate partner violence and women’s physical and mental health in the WHO multi-country study on women’s health and domestic violence: an observational study. Lancet.

[CR25] Ferguson CJ, Dyck D (2012). Paradigm change in aggression research: the time has come to retire the general aggression model. Aggress Violent Behav.

[CR26] Ferguson CJ, Cruz AM, Martinez D, Rueda SM, Ferguson DE, Negy C (2008). Personality, parental, and media influences on aggressive personality and violent crime in young adults. J Aggress Maltreat Trauma.

[CR27] Ferguson CJ, Rueda SM, Cruz AM, Ferguson DE, Fritz S, Smith SM (2008). Violent video games and aggression causal relationship or byproduct of family violence and intrinsic violence motivation?. Crim Justice Behav.

[CR28] Ferguson CJ, Ivory JD, Beaver KM (2013). Genetic, maternal, school, intelligence, and media use predictors of adult criminality: a longitudinal test of the catalyst model in adolescence through early adulthood. J Aggress Maltreat Trauma.

[CR29] Fulu E, Jewkes R, Roselli T, Garcia-Moreno C (2013). Prevalence of and factors associated with male perpetration of intimate partner violence: findings from the UN multi-country cross-sectional study on men and violence in Asia and the pacific. Lancet Glob Health.

[CR30] Golden SD, Perreira KM, Durrance CP (2013). Troubled times, troubled relationships: how economic resources, gender beliefs, and neighborhood disadvantage influence intimate partner violence. J Interpers Violence.

[CR31] Halliday Hardie J, Lucas A (2010). Economic factors and relationship quality among young couples: comparing cohabitation and marriage. J Marriage Fam.

[CR32] Harris KM (2013). The add health study: design and accomplishments.

[CR33] Harris KM, Halpern C, Whitsel E, Hussey J, Tabor J, Entzel P (2009). The national longitudinal study of adolescent health: research design.

[CR34] Khalifeh H, Hargreaves J, Howard LM, Birdthistle I (2013). Intimate partner violence and socioeconomic deprivation in England: findings from a national cross-sectional survey. Am J Public Health.

[CR35] Kim JC, Watts CH, Hargreaves JR, Ndhlovu LX, Phetla G, Morison LA (2007). Understanding the impact of micrrofinance-based intervention on women’s employerment and the reduction of intimate partner violence in South Africa. Am J Public Health.

[CR36] Langhinrichsen-Rohling J, McCullars A, Misra TA (2012). Motivations for men and women’s intimate partner violence perpetration: a comprehensive review. Partner Abuse.

[CR37] Langhinrichsen-Rohling J, Selwyn C, Rohling M (2012). Rates of bidirectional versus unidirectional intimate partner violence across samples, sexual orientations, and race/ethnicities: a comprehensive review. Partner Abuse.

[CR38] Liu Y, Njai RS, Greenlund KJ, Chapman DP, Croft JB (2014). Relationships between housing and food insecurity, frequent mental distress, and insufficient sleep among adults in 12 US states, 2009. Prev Chronic Dis.

[CR39] Lovallo WR (2013). Early life adversity reduces stress reactivity and enhances impulsive behavior: Implications for health behaviors. Int J Psychophysiol.

[CR40] Mason B, Smithey M (2012). The effects of academic and interpersonal stress on dating violence among college students a test of classical strain theory. J Interpers Violence.

[CR41] Matjasko JL, Niolon PH, Valle LA (2013). The role of economic factors and economic support in preventing and escaping from intimate partner violence. J Policy Anal Manage.

[CR42] Neff JA, Holamon B, Schluter TD (1995). Spousal violence among Anglos, Blacks, and Mexican Americans: the role of demographic variables, psychosocial predictors, and alcohol consumption. J Fam Violence.

[CR43] Okuda M, Olfson M, Hasin D, Grant BF, Lin K-H, Blanco C (2011). Mental health of victims of intimate partner violence: results from a national epidemiologic survey. Psychiatr Serv.

[CR44] Postmus JL, Plummer S-B, McMahon S, Murshid NS, Kim MS. Understanding economic abuse in the lives of survivors. J Interpers Violence. 2011: doi:10.1177/0886260511421669. 10.1177/0886260511421669 21987509

[CR45] Próspero M (2007). Mental health symptoms among male victims of partner violence. Am J Mens Health.

[CR46] Próspero M, Kim M (2009). Mutual partner violence mental health symptoms among female and male victims in four racial/ethnic groups. J Interpers Violence.

[CR47] Renner LM, Schwab-Reese L, Peek-Asa C, Ramirez M (2015). Reporting patterns of unidirectional and bidirectional verbal aggression and physical violence among rural couples. J Fam Violence.

[CR48] Roberts AL, McLaughlin KA, Conron KJ, Koenen KC (2011). Adulthood stressors, history of childhood adversity, and risk of perpetration of intimate partner violence. Am J Prev Med.

[CR49] Ruel E, Hauser RM (2013). Explainging the gender wealth gap. Demography.

[CR50] Shorey RC, Sherman AE, Kivisto AJ, Elkins SR, Rhatigan DL, Moore TM (2011). Gender differences in depression and anxiety among victims of intimate partner violence: the moderating effect of shame proneness. J Interpers Violence.

[CR51] Shortt JW, Capaldi DM, Kim HK, Tiberio SS (2013). The interplay between interpersonal stress and psychological intimate partner violence over time for young at-risk couples. J Youth Adolesc.

[CR52] Shrier I, Platt RW (2008). Reducing bias through directed acyclic graphs. BMC Med Res Methodol.

[CR53] Slep AMS, Foran HM, Heyman RE, Snarr JD (2010). Unique risk and protective factors for partner aggression in a large scale Air Force survey. J Community Health.

[CR54] Straus H, Cerulli C, McNutt LA, Rhodes KV, Conner KR, Kemball RS (2009). Intimate partner violence and functional health status: associations with severity, danger, and self-advocacy behaviors. J Womens Health.

[CR55] Whitaker MP. Motivational attributions about intimate partner violence among male and female perpetrators. J Interpers Violence. 2013; doi:10.1177/0886260513505211. 10.1177/0886260513505211 24097910

[CR56] Yvas S, Watts C (2009). How does economic empowerment affect women’s risk of intimate partner violence in low and middle income countries? A systematic review of published evidence. J Int Dev.

